# Supplementary material for: The Small GTPase Ran Increases Sensitivity of Ovarian Cancer Cells to Oncolytic Vesicular Stomatitis Virus
Source: Pharmaceuticals (Basel). 2024 Dec 10;17(12):1662. doi: 10.3390/ph17121662 (PMC11677601; doi:10.3390/ph17121662)
Supplement: Supplementary file 1 [file pharmaceuticals-17-01662-s001.zip › pharmaceuticals-3327500-supplementary.pdf]

TABLE S1

Cell line	Cancer subtype	Prior Treatment	Impact of oVSV on RanGTP
TOV3133G	HGSOC	Naive	No impact
TOV2414	Mucinous carcinoma	Naive	
TOV1946	HGSOC	Naive	
TOV2835EP	HGSOC	PTX/CBP	Decrease in Ran activation
TOV3392D	Clear cell carcinoma	5FU, EPI, CPP, trastuzumab	
TOV112D	Endometrioid carcinoma	Naive	
TOV3041G	HGSOC	PTX/CBP	Increase in Ran activation
OV3331	Undifferenciated adenocarcinoma	PTX/CBP, epothilone	
TOV21G	Clear cell carcinoma	Naive	
OV1946	HGSOC	Naive	
OV2085	HGSOC	PTX/CBP	

Table 1. Characteristics of the ovarian cancer cell lines used in this study.

Cancer subtypes, previous treatments and impact of oVSV on RanGTP (as measured by Ran activation assays in Fig. 3). Nomenclature of the cell lines: derived from ascites (OV) or primary tumor tissue (TOV) from the left (G) or right (D) ovary. HGSOC = high grade serous ovarian cancer, PTX = paclitaxel, CBP = carboplatin, 5-FU = 5-fluorouracil, EPI = epirubicin, CPP = cyclophosphamide.

FIGURE S1

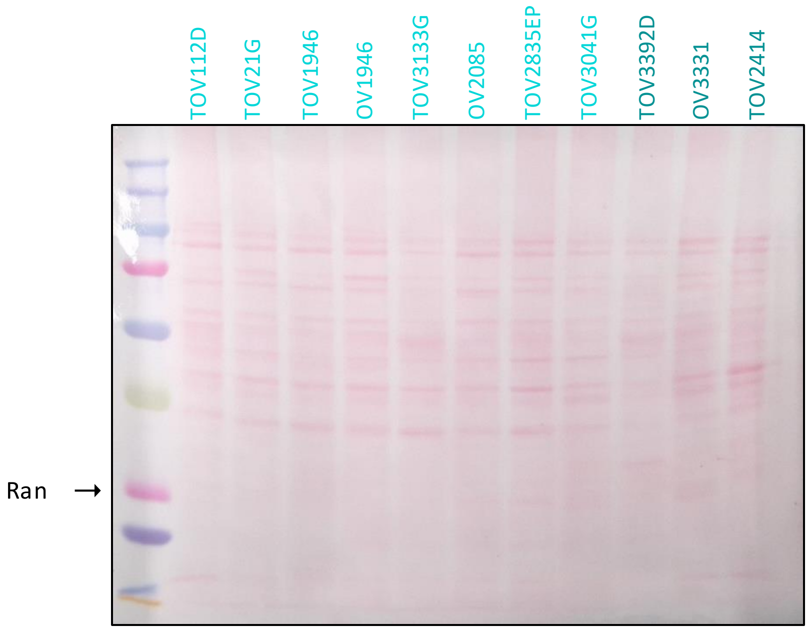

Figure S1. Comparable protein levels are found in all cell lines from Fig. 1C.

Picture of nitrocellulose membrane stained with ponceau red. Ran protein (25kDa) is indicated.

FIGURE S2

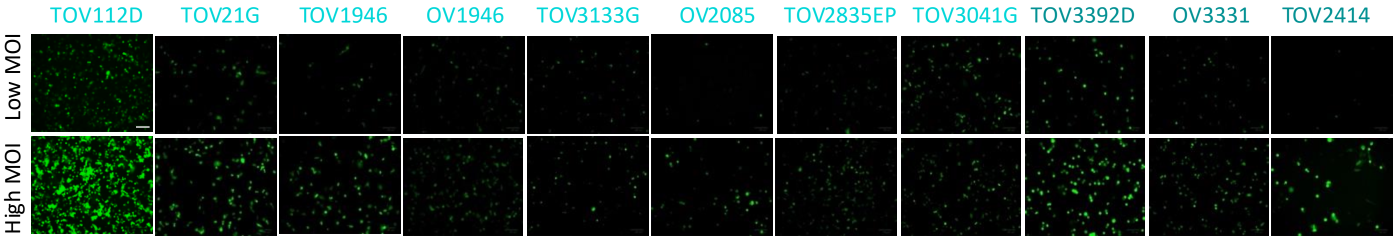

Figure S2. Infection of EOC cells with VSVΔ51.

Fluorescence microscopy pictures of EOC cells (OV2085, OV1946, TOV112D, TOV1946, TOV21G, TOV3392D, TOV2835EP, TOV3041G, OV3331, TOV2414 and TOV3133G) infected for 24h at different MOIs with VSVΔ51. Cell lines were infected with low and high MOIs (1 and 10 respectively for OV2085, OV1946, TOV112D, TOV1946 and TOV21G; 10 and 100 respectively for TOV3392D, TOV2835EP, TOV3041G, OV3331, TOV2414 and TOV3133G). Scale bar = 100µm.

FIGURE S3

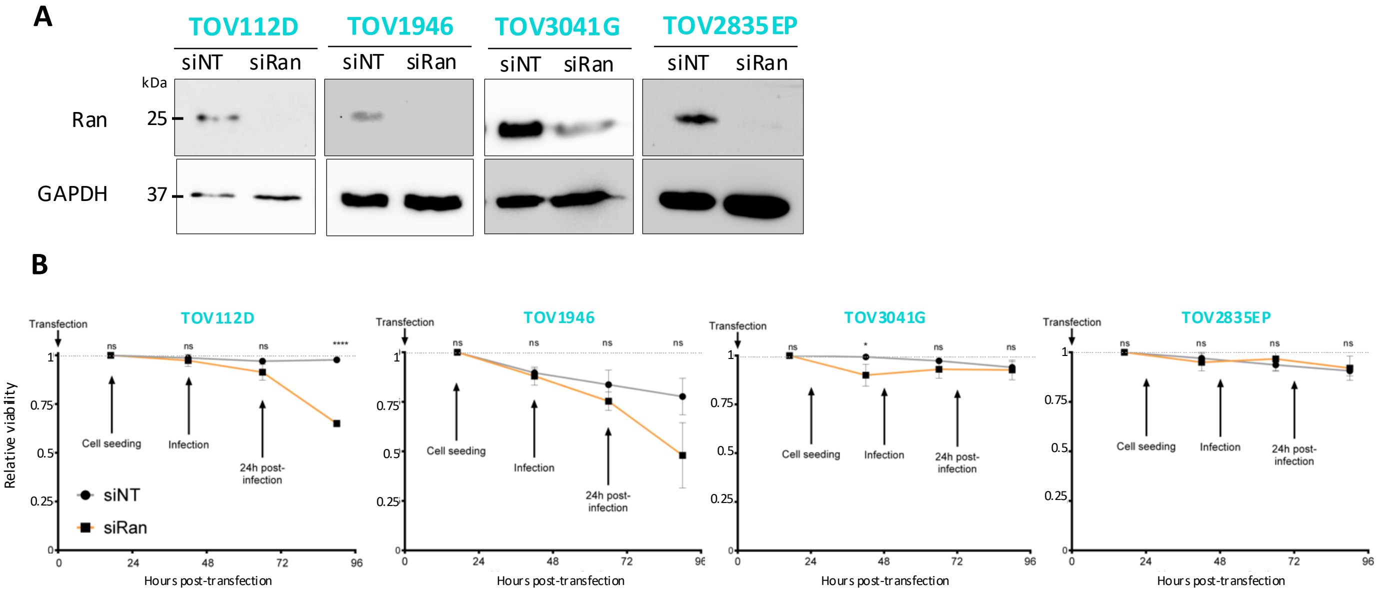

Figure S3. Ran siRNA-transfected cells are viable at timepoints of sample collection.

TOV112D, TOV1946, TOV3041G and TOV2835EP cells were transfected with control or Ran-targeting siRNAs. **A**) western blot analysis confirmed knock-down for **B**) Cell viability was measured by trypan blue at different time points post-transfection for each cell line. Samples were not infected, but arrows represent the corresponding infection and sample collection time points from Fig 2. Unpaired multiple t-test (n=3): ns: $p > 0.05$; *: $p \leq 0.05$; **: $p \leq 0.01$; ***: $p \leq 0.001$; ****: $p \leq 0.0001$.

FIGURE S4

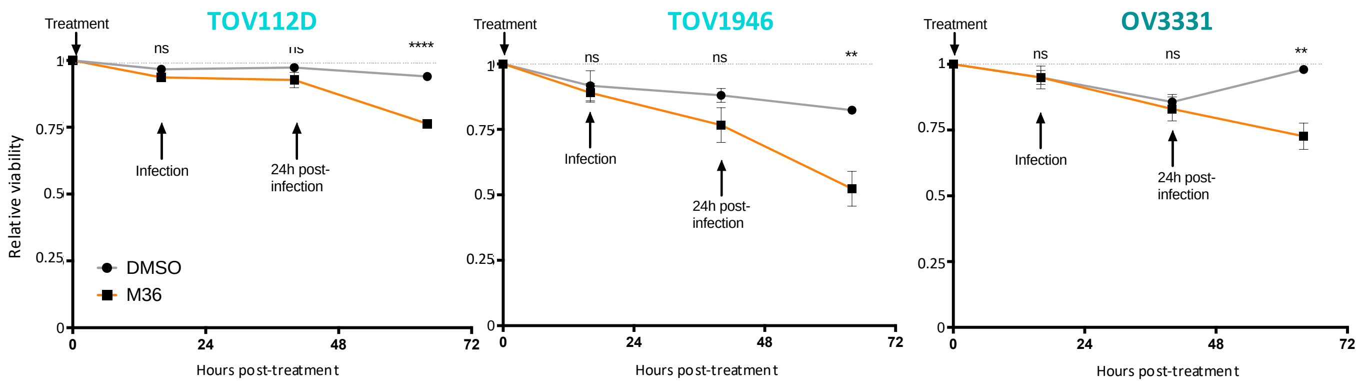

Figure S4. M36-treated cells remain viable at the experimental timepoint analyzed.

Cell viability of TOV112D, TOV1946 and OV3331 cells measured at different time points post-M36 treatment. Samples were not infected, and arrows represent the corresponding infection and sample collection time points from Fig 3. Unpaired multiple t-test (n=3): ns: $p > 0.05$; *: $p \leq 0.05$; **: $p \leq 0.01$; ***: $p \leq 0.001$; ****: $p \leq 0.0001$.

FIGURE S5

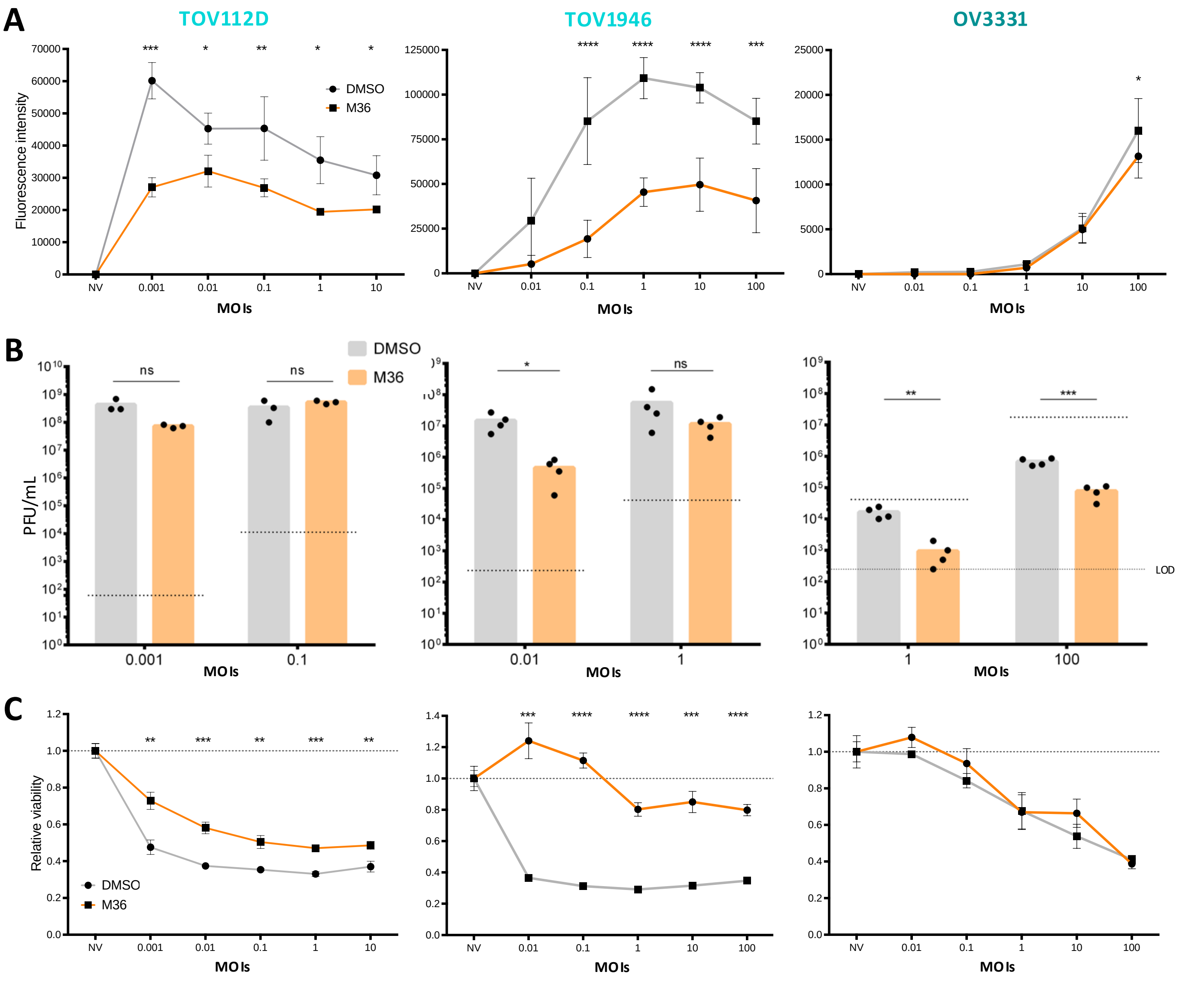

Figure S5. Ran inhibition concomitant to VSV infection impairs its replication and cancer killing abilities.

Conditions are identical as in Figure 4 except that M36 treatment and infection occurred at the same time. After 24h of infection, **A**) fluorescent signal was quantified as well as **B**) viral outputs and **C**) cell viability. Unpaired multiple t-test (n≥3): ns: $p > 0.05$; *: $p \leq 0.05$; **: $p \leq 0.01$; ***: $p \leq 0.001$; ****: $p \leq 0.0001$.
